# One-transistor static random-access memory cell array comprising single-gated feedback field-effect transistors

**DOI:** 10.1038/s41598-021-97479-x

**Published:** 2021-09-09

**Authors:** Sangik Choi, Jaemin Son, Kyoungah Cho, Sangsig Kim

**Affiliations:** 1 Department of Semiconductor Systems Engineering, Korea University, 145 Anam-ro, Seongbuk-gu, Seoul, 02841 Republic of Korea; 2 Department of Electrical Engineering, Korea University, 145 Anam-ro, Seongbuk-gu, Seoul, 02841 Republic of Korea

**Keywords:** Electronic devices, Electrical and electronic engineering

## Abstract

In this study, we fabricated a 2 × 2 one-transistor static random-access memory (1T-SRAM) cell array comprising single-gated feedback field-effect transistors and examined their operation and memory characteristics. The individual 1T-SRAM cell had a retention time of over 900 s, nondestructive reading characteristics of 10,000 s, and an endurance of 10^8^ cycles. The standby power of the individual 1T-SRAM cell was estimated to be 0.7 pW for holding the “0” state and 6 nW for holding the “1” state. For a selected cell in the 2 × 2 1T-SRAM cell array, nondestructive reading of the memory was conducted without any disturbance in the half-selected cells. This immunity to disturbances validated the reliability of the 1T-SRAM cell array.

## Introduction

Over the years, various studies have been conducted to overcome the issues posed by the size and power consumption of conventional static random-access memory (SRAM)^1–4^. Decreasing the number of component transistors in conventional SRAM cells has been suggested as a suitable way to combat these problems^5–13^. However, reduction in the operation power of SRAM cells remains a challenging issue. This is mainly because the low-power operation of SRAM cell arrays decreases the reliability of memory operation due to disturbances caused by the half selected cell^14–16^.

More recently, simulation studies have demonstrated that the low standby power consumption and reliability of one-transistor SRAM (1T-SRAM) cells, each of which consists of a single-gated feedback field-effect transistor (FBFET), provide a promising possibility for the future of memory devices^17^. Compared with metal–oxide–semiconductor field-effect transistors (MOSFETs) conventionally used in SRAM cells, FBFETs, operating with a positive feedback loop mechanism, exhibit an extremely low subthreshold swing (~ 0 mV/dec), high on/off current ratios, and bi-stable states^18–22^. Moreover, the use of 1T-SRAM cells reduces usage area on chips when compared with conventional 6T-SRAM cells constructed with MOSFETs. Hence, FBFETs are more advantageous than MOSFETs in terms of usage in SRAM cells, thus increasing performance while reducing their size. To apply our 1T-SRAM cell to computing systems, the reliability of the cell array should be confirmed. Hence, in this study, we fabricated a 2 × 2 1T-SRAM cell array using four FBFETs and confirmed its immunity against half-selected cell disturbances during nondestructive reading of the memory.

## Experimental section

**Figure 1:**
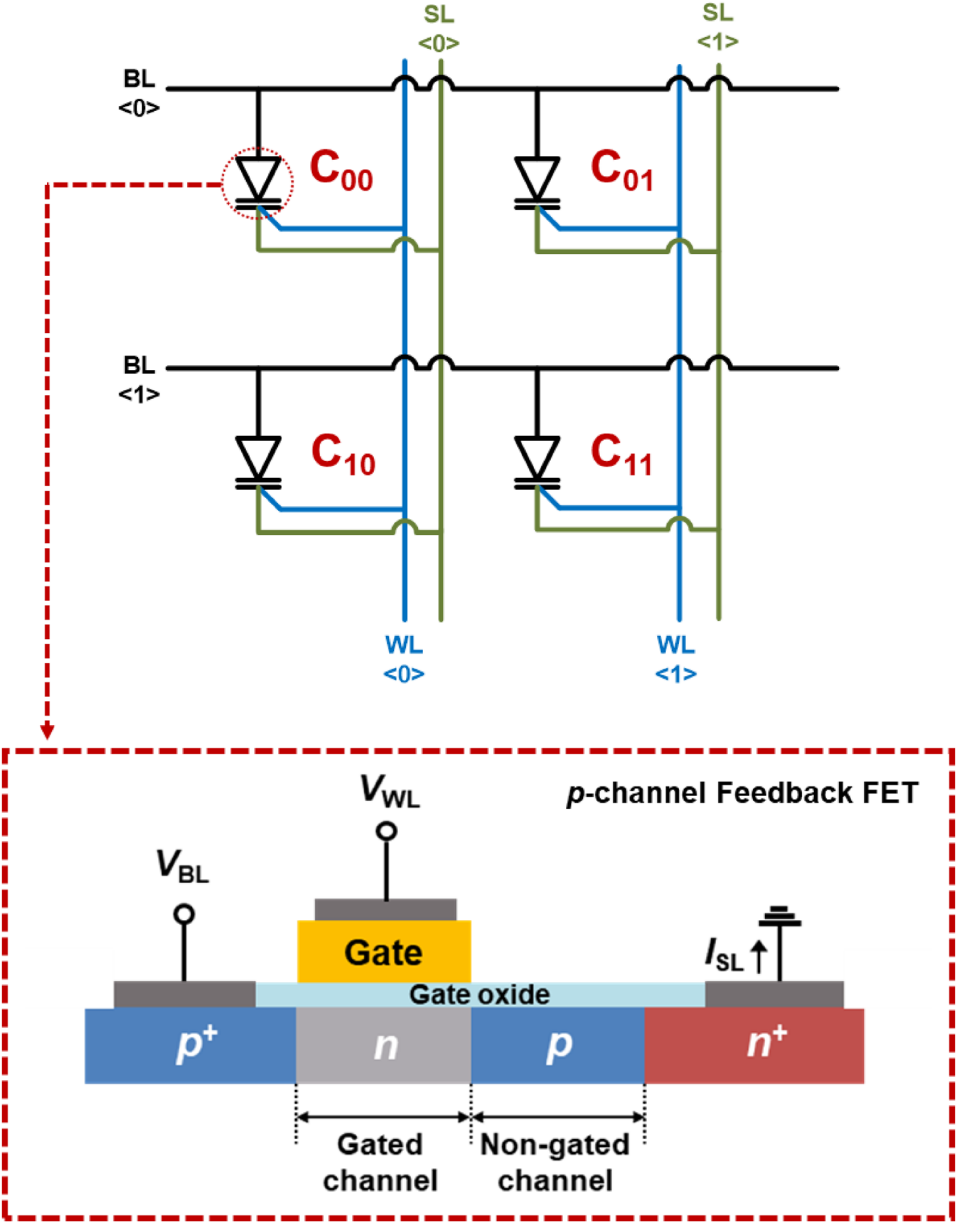
Schematics of a 2 × 2 array of 1T-SRAM cells and a *p*-channel FBFET.

Figure 1 shows the schematic of a 2 × 2 1T-SRAM cell array consisting of four *p*-channel FBFETs with a *p*^+^-*n*-*p*-*n*^+^ structure and with each channel (gated or non-gated) being 1.5 μm in length. Herein, four *p*-channel FBFETs stand for four SRAM cells because only one transistor performs the function of a SRAM cell in our device structure. The 1T-SRAM cells were fabricated on an SOI wafer with 2-μm-thick buried oxide through a CMOS-compatible process. First, a 340 nm silicon active layer was prepared using stepper photolithography and an anisotropic dry etching process. The *n*-well was formed by the implantation of P^+^ ions at a dose of 3 × 10^13^ cm^−2^ with an ion energy of 60 keV, annealed at 1100 °C for 30 min. The *p*-type channel region was formed by the implantation of BF_2_^+^ ions at a dose of 6 × 10^13^ cm^−2^ with an ion energy of 40 keV. For the formation of the *n*^+^ source and *p*^+^ drain regions, P^+^ and BF_2_^+^ ions were implanted at a dose of 3 × 10^15^ cm^−2^ using a masked ion implantation method at ion energies of 100 keV and 30 keV, respectively. A 22-nm-thick SiO_2_ gate dielectric was thermally grown at 850 °C for 300 min, before a 400-nm-thick poly-Si gate was formed using low-pressure chemical vapor deposition, photolithography, and a dry etching process. Finally, a Ti/TiN/Al/TiN metal alloy was deposited for the bit line (BL), word line (WL), and source line (SL) contacts. The 2 × 2 1T-SRAM cell array was completed with shared BLs horizontally and shared WLs and SLs vertically.

The electrical characteristics were measured at room temperature using a semiconductor parameter analyzer (HP4155C, Agilent), a Tektronix AFG 31,000 arbitrary function generator, a Tektronix MDO3054 mixed-domain oscilloscope, and a Keithley 2636 B sourcemeter SMU instrument. The limits of the retention and endurance measurement were 900 s and 10^8^ cycles, respectively.

## Results and discussion

**Figure 2:**
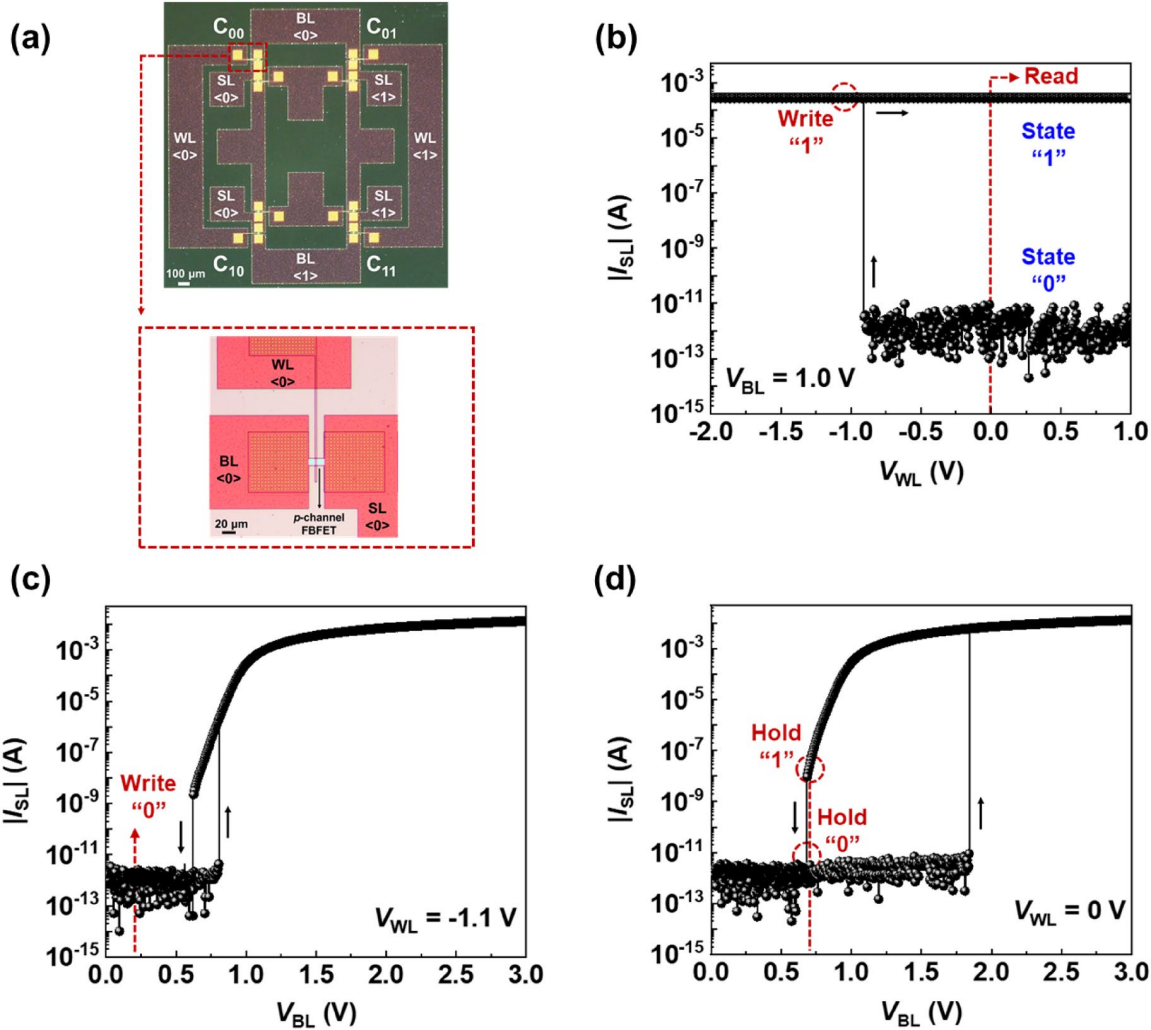
(**a**) Optical image of a 2 × 2 1T-SRAM cell array and a *p*-channel FBFET. (**b**) Transfer function of a selected 1T-SRAM cell at *V*_BL_ = 1.0 V. Output curves of the 1T-SRAM cell at (**c**) *V*_WL_ =  − 1.1 V and (**d**) *V*_WL_ = 0.0 V.

The optical images in Fig. 2a show our 2 × 2 1T-SRAM cell array consisting of four cells (i.e., C_00_, C_01_, C_10_, and C_11_), where C_00_ shares BL <0> and WL <0> with C_01_ and C_10_, while C_11_ shares WL <1> and BL <1> with C_01_ and C_10_. The transfer function in Fig. 2b shows the bistable states for an individual 1T-SRAM cell. The abrupt increase in |*I*_SL_| when *V*_WL_ reached − 0.91 V in the transfer function indicates the latch-up phenomenon, which induces a positive feedback loop. Herein, negative *V*_WL_ and positive *V*_BL_ are responsible for barrier-height modulation. Carrier accumulation in the potential wells of the conduction and valence bands in the channels of the FBFET determine the current state. Our 1T-SRAM cell uses holes as the majority charge carriers for the positive feedback loop. The “0” and “1” states are described by the absence and presence of excess charge carriers in the potential wells, respectively. For the write “1” (W1) operation, *V*_WL_ was selected to be − 1.1 V, which is below the latch-up voltage of − 0.91 V. The read “1” and “0” (R1, R0) operations were carried out at *V*_WL_ = 0.0 V, and R0 and R1 states are triggered before and after the W1 operation. The hysteresis characteristics of the output functions for each individual 1T-SRAM cell are shown in Fig. 2c,d. These curves are used to determine the remaining operations. *V*_BL_ was increased in a forward sweep from 0 to 3 V, |*I*_SL_| abruptly increased at two points, at a *V*_BL_ of 0.81 V for *V*_WL_ =  − 1.1 V and at a *V*_BL_ of 1.85 V for *V*_WL_ = 0 V. However, during the reverse sweep, |*I*_SL_| fell abruptly at a *V*_BL_ of 0.68 V, which indicates the latch-down phenomenon. For the operations of the 1T-SRAM cell, *V*_BL_ was selected to be 0.2 V for the write “0” (W0) operation and 0.68 V for the hold “1” and “0” (H1, H0) operations.

**Figure 3:**
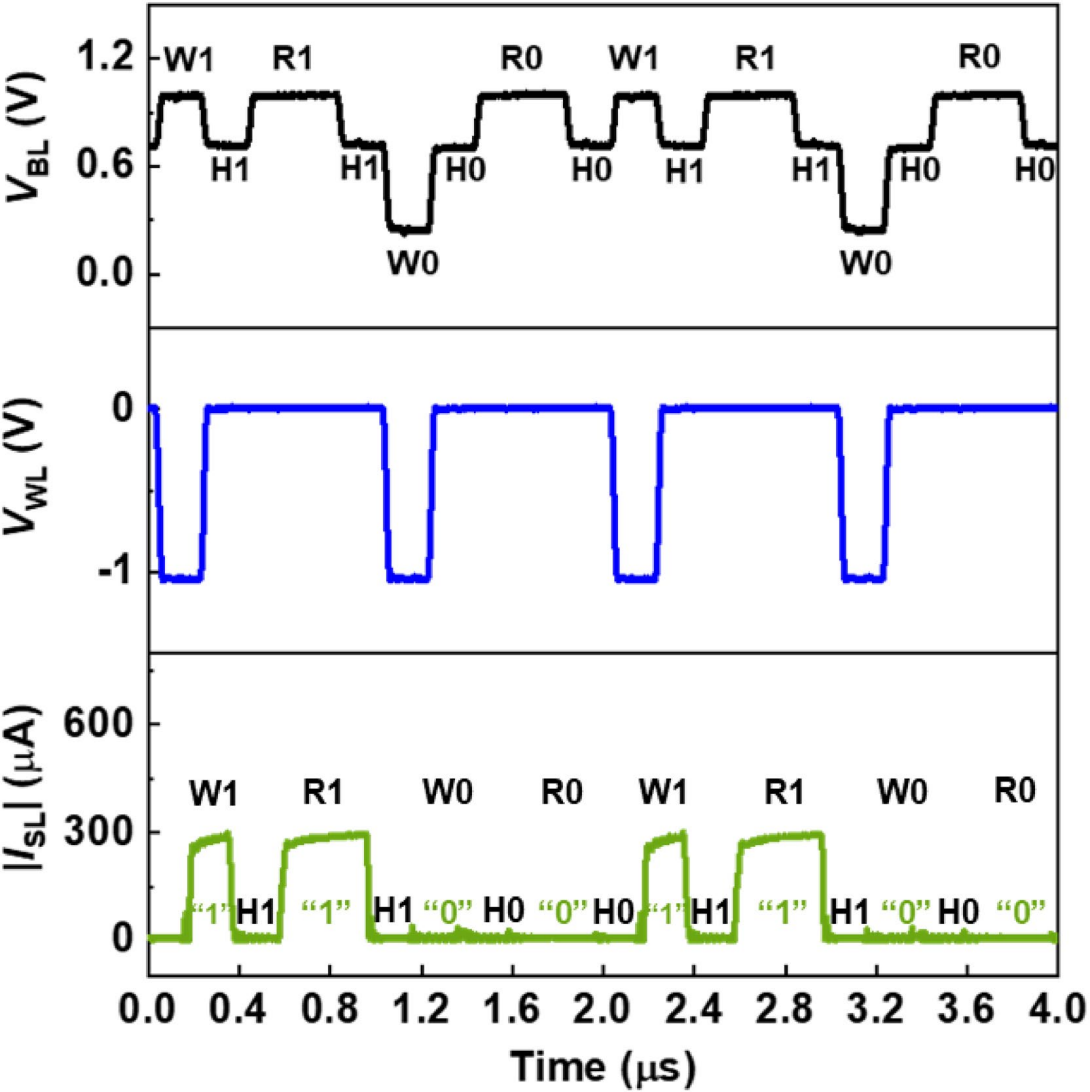
Timing diagrams of 1T-SRAM cell memory operations. The pulse width of the write and hold operations was 200 ns, and the pulse width of the read operation was 400 ns.

A timing diagram for a sequence of the write, hold, and read operations of the 1T-SRAM cell is shown in Fig. 3. Here, the W1, W0, H1, and H0 operations were performed for 200 ns, and the R1 and R0 operations were performed for 400 ns. The operating conditions are presented in Table 1. A delay time of 140 ns in |*I*_SL_| was inherent in the measurement system; nevertheless, our 1T-SRAM cell worked without any malfunction. The cycle starts after the H1 operation, and during the R1 operation, |*I*_SL_| was 300 μA, indicating the “1” state. Next, the W0 operation was performed at a *V*_BL_ of 0.2 V and at a *V*_WL_ of − 1.1 V, at which point |*I*_SL_| became ~ 1 pA. The decreases in *V*_BL_ and negative *V*_WL_ are responsible for breaking the positive feedback loop. After the H0 operation, the R0 operation |*I*_SL_| was kept at ~ 1 pA, indicating the “0” state. Although *V*_BL_ and *V*_WL_ are the same for the R0 and R1 operations, there is a differing |*I*_SL_| value, which is determined by the previous W0 or W1 |*I*_SL_| values, respectively. |*I*_SL_| was ~ 1 pA for the H0 operation and ~ 9 nA for the H1 operation. The magnitude of |*I*_SL_| was used to calculate the standby power consumption. The standby power consumption was 0.7 pW for the “0” state and 6 nW for the “1” state. In this regard, the standby power consumption of our 1T-SRAM cell is superior to other SRAM cells^23–26^.

**Table 1:** Operating conditions of the 1T-SRAM cell.

Voltage	Write “1”	Write “0”	Read	Hold
*V*_BL_ (V)	1.0	0.2	1.0	0.68
*V*_WL_ (V)	− 1.1	− 1.1	0.0	0.0

**Figure 4:**
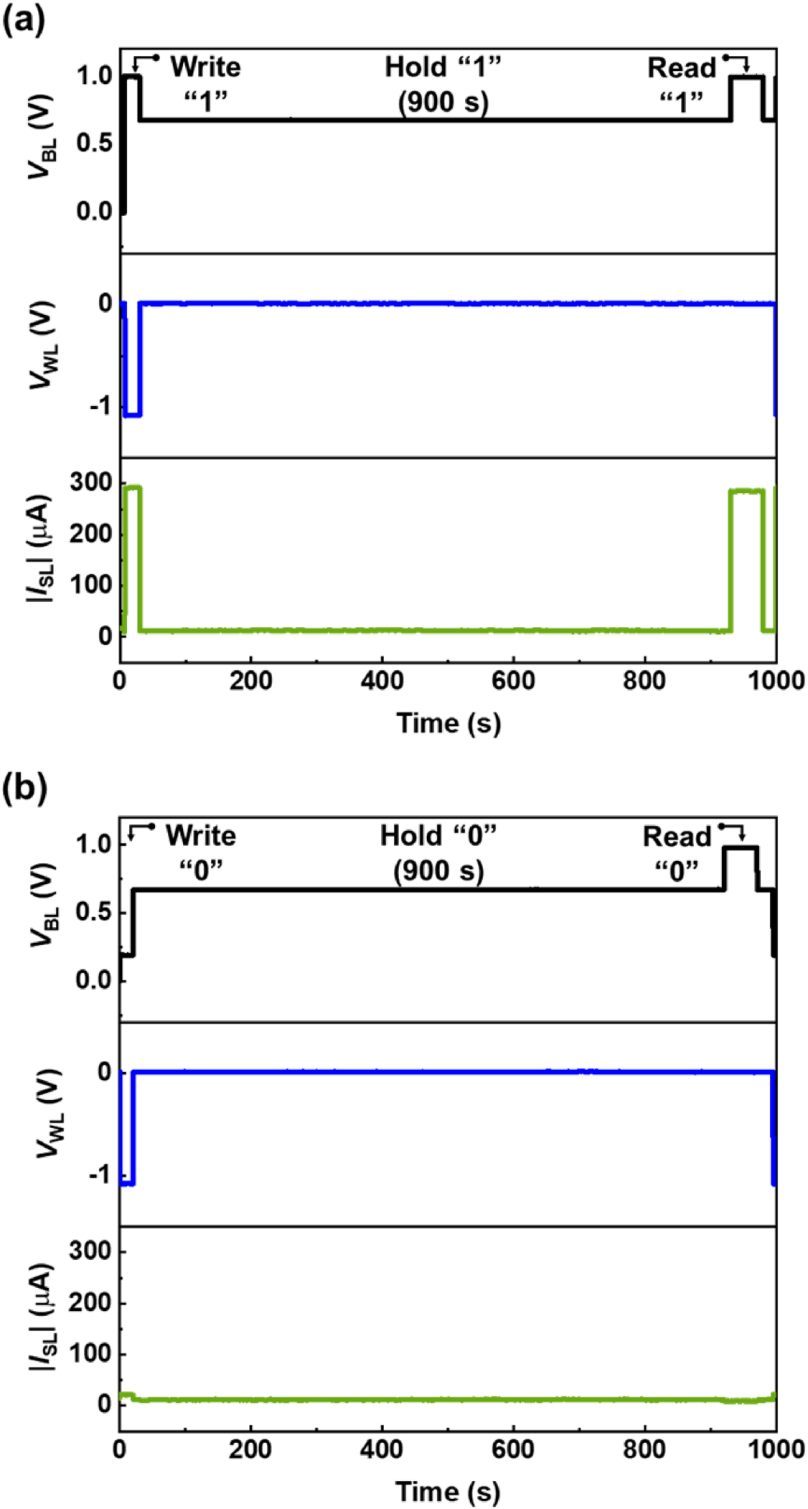
Retention characteristics of (**a**) the “1” state and (**b**) the “0” state.

Figures 4 show the retention characteristics of the “1” (a) and “0” (b) states after a holding time of 900 s. During the H1 operation, there was no loss in charge carriers accumulated in the channel; consequently, |*I*_SL_| in the R1 operation was 300 μA, which is the same as that of the W1 operation. On the other hand, the absence of charge carriers in the potential well during the W0 operation produced |*I*_SL_| of 1 pA during the H0 operation. This |*I*_SL_| became the output signal for the R0 operation. The detection limit during experimentation was 900 s; this was mainly due to the test equipment constraints and the memory retention of the 1T-SRAM cell is much longer.

**Figure 5:**
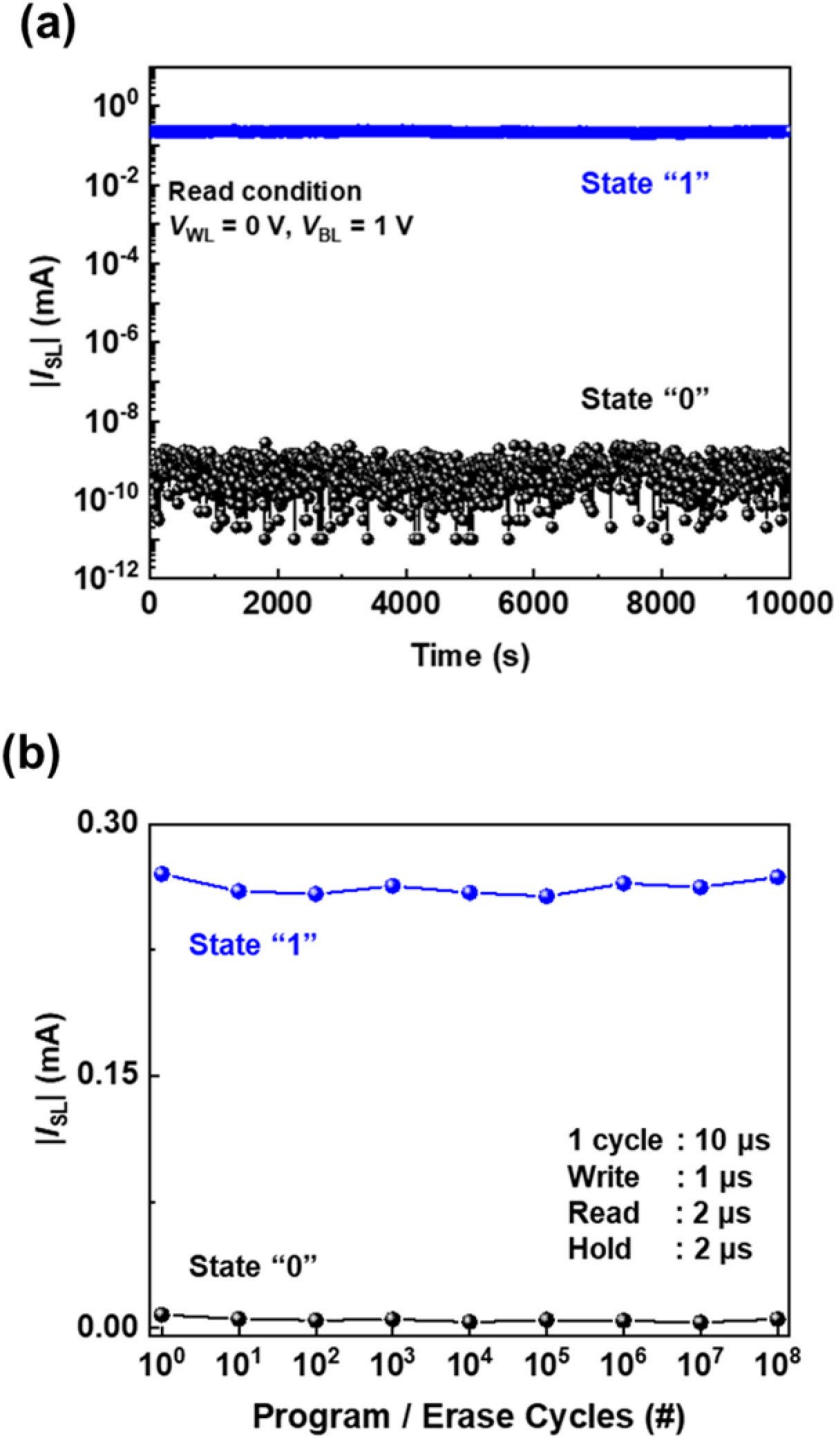
For 1T-SRAM cell: (**a**) nondestructive reading characteristics at *V*_WL_ = 0.0 V and *V*_BL_ = 1.0 V and (**b**) endurance characteristics.

The nondestructive reading characteristics of the 1T-SRAM cell shown in Fig. 5a reveals that the “1” and “0” states were maintained for 10,000 s, with a sensing margin of 10^9^, when |*I*_SL_| was under the read conditions of *V*_WL_ = 0 V and *V*_BL_ = 1 V. These excellent nondestructive reading characteristics show that the states are not destroyed during the reading operation even over an extended period and there is no degradation associated with the accumulation of charge carriers in the channel region. The endurance of the 1T-SRAM cell shown in Fig. 5b indicates the maintenance of the “0” and “1” states even after 10^8^ cycles where one cycle consists of the program and erase operations. The |*I*_SL_| magnitude in the “0” state is a few μA being the detection limit of our measurement system so that the sensing margin for the endurance becomes 10^2^. Considering these criteria, our 1T-SRAM cell can be considered a reliable memory device.

**Figure 6:**
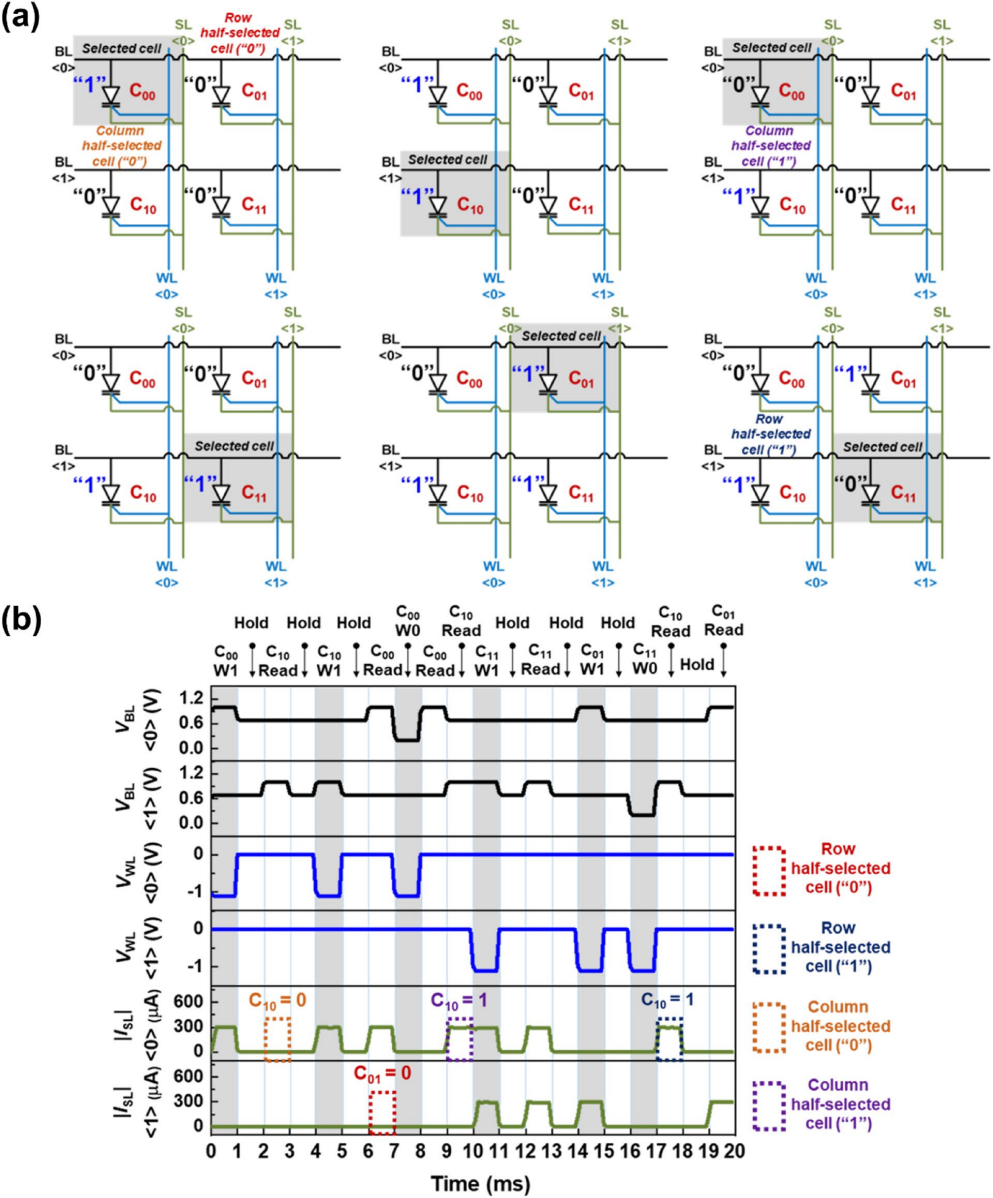
For 2 × 2 1T-SRAM cell array: (**a**) schematic diagram of the memory operations and (**b**) timing diagrams.

To further examine the reliability of the 2 × 2 1T-SRAM cell array, memory operations were performed to monitor the half-selected cells, as shown in Fig. 6. For example, when C_00_ was selected, C_01_ and C_10_ became the row and column half-selected cells respectively, as shown in Fig. 6a. The operations of the row and column half-selected cells are influenced by BL and WL disturbances, respectively. Figure 6b shows the timing diagrams of the array. The W1 operation at C_00_ was carried out at a *V*_BL<0>_ of 1.0 V, *V*_WL<0>_ of − 1.1 V, and an |*I*_SL_|_<0>_ of 300 μA which produces the “1” state output. When the read operation at C_10_, which shares WL <0> with C_00_, was performed after the hold operation, |*I*_SL_|_<0>_ indicates the output signal was in the “0” state. This demonstrates that there was no disturbance in the column half-selected cell. For the read operation at C_01_, which shares BL <0> with C_00_, |*I*_SL_|_<1>_ indicates the output signal was in the “0” state, implying immunity against disturbance in the half-selected cell rows. Thus, there was no disturbance in half-selected cells for both the rows and columns when C_00_ was in the “1” state. In addition, the states of the column and row half-selected cells for C_00_ in the “0” state were unchanged even after the W1 operation at C_00_. Moreover, the immunity against disturbance in the half-selected cells in the “0” state was reaffirmed by the read operation at C_10_ after the W0 operation at C_11_ while having a shared BL <0> line. Our 1T-SRAM cell array demonstrates reliability in nondestructive readout operations. Considering that only one transistor performs the function of a SRAM cell, our SRAM cells have advantages of lower power operation and reduced size, compared to conventional SRAM cells.

## Conclusions

In this study, we fabricated a 2 × 2 1T-SRAM cell array and investigated its operation and memory characteristics. An individual 1T-SRAM cell consumed 0.7 pW for holding the “0” state and 6 nW for holding the “1” state. The cells had a retention time of over 900 s, nondestructive reading characteristics of 10,000 s, and an endurance of 10^8^ cycles. Furthermore, nondestructive memory reading operations were conducted without any disturbance to the half-selected cells validating the reliability of the array. The size and power consumption of our 1T-SRAM cell array means that it can be used for computing systems that require a large amount of on-chip memory.
